# Toxicokinetic–Toxicodynamic Model to Assess Thermal Stress

**DOI:** 10.1021/acs.est.3c05079

**Published:** 2023-12-08

**Authors:** Annika Mangold-Döring, Jan Baas, Paul J. van den Brink, Andreas Focks, Egbert H. van Nes

**Affiliations:** † Department of Aquatic Ecology and Water Quality Management, Wageningen University and Research, P.O. Box 47, 6700 AA Wageningen, The Netherlands; ‡ Wageningen Environmental Research, P.O. Box 47, 6700 AA Wageningen, The Netherlands; § System Science Group/Institute of Mathematics, Osnabrück University, Barbarastrasse 12, D-49076 Osnabrück, Germany

**Keywords:** temperature stress, TK–TD models, environmental risk assessment

## Abstract

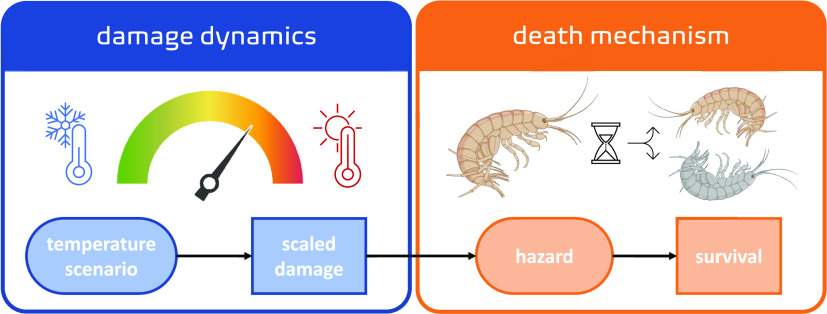

Temperature is a crucial environmental factor affecting the distribution and performance of ectothermic organisms. This study introduces a new temperature damage model to interpret their thermal stress. Inspired by the ecotoxicological damage model in the General Unified Threshold model for Survival (GUTS) framework, the temperature damage model assumes that damage depends on the balance between temperature-dependent accumulation and constant repair. Mortality due to temperature stress is driven by the damage level exceeding a threshold. Model calibration showed a good agreement with the measured survival of *Gammarus pulex* exposed to different constant temperatures. Further, model simulations, including constant temperatures, daily temperature fluctuations, and heatwaves, demonstrated the model’s ability to predict temperature effects for various environmental scenarios. With this, the present study contributes to the mechanistic understanding of temperature as a single stressor while facilitating the incorporation of temperature as an additional stressor alongside chemicals in mechanistic multistressor effect models.

## Abstract 2

We introduce a novel temperature damage model, enhancing the understanding of temperature effects on organisms and aiding the integration of temperature as a stressor in process-based effect models for environmental risk assessment.

## 1 Introduction

As a factor shaping the environmental niche for organisms, temperature influences the distribution of species around the globe.^1^ For ectotherms, i.e., organisms whose regulation of body temperature depends on external drivers, the relationship of their distribution with temperature is often defined via the organisms’ thermal tolerances and performance, constrained by, i.e., lower and upper temperature limits.^2−4^ All internal processes depend on temperature, and it is often found that respiration and other processes are exponentially increasing with temperature.^5−7^ At the limits of species’ thermal tolerances, temperature becomes a stress factor that can lead to mortality.^2,8^ Next to the thermal limits, species’ sensitivity to other environmental factors determines their performance and survival in the environment.^9^ From an ecotoxicological perspective, it is particularly interesting to investigate how temperature changes projected due to climate change will influence species’ sensitivity to pesticides or other chemicals in the environment. Indeed, there have recently been a variety of studies looking at those interactions, which found evidence for an increase in toxicity by increasing temperature conditions.^10−14^ This highlights the need to address future climate scenarios in environmental risk assessment (ERA) for chemicals.^15^

A promising way to incorporate temperature scenarios in ERA is the implementation of the influence of temperature in mechanistic or process-based models such as toxicokinetic–toxicodynamic (TK–TD) models. TK–TD models enable extrapolation to realistic environmental circumstances (i.e., time-variable exposure patterns) and give mechanistic insight into the TK (i.e., uptake and elimination of the chemical) and TD processes (i.e., through the damage concept). However, temperature may affect the inherent sensitivity of an organism to chemical exposure (i.e., influence the effect threshold represented in the TD processes) or the kinetics of the chemical (i.e., TK processes). Thus, it is essential to include temperature in TK–TD approaches to interpret the observed effects correctly. There are two ways to account for the influence of temperature on an individual’s response to chemical stress. First, temperature can be interpreted as a modulating factor influencing the TK–TD processes of the chemical.^16,17^ Second, temperature can be approached as a stressor by itself, in addition to the chemical stress. Even though, in reality, it might be a combination of both, looking at these two different approaches will help us to understand the mechanisms of the combined stressors, chemicals, and temperature.

Although the influence of temperature on organisms has been investigated frequently,^18,19^ it seems to be challenging to derive general models to quantitatively describe those thermal responses.^20^ In biology, the most used model to account for temperature influences is the empirical Arrhenius equation,^21^ although its limitations have been discussed.^22−24^ Most importantly, the Arrhenius equation fits only the exponentially increasing parts of the temperature response relationship, while in reality, an optimum effect is more likely.^4^ For example, the nonmonotonous effect of temperature can be observed in daphnids, where the reproduction is highest between 15 and 20 °C and decreases significantly below and above these temperatures.^25^

Experimentally, the temperature tolerance of aquatic species is commonly assessed in static or dynamic assays, determining the temperature performance curves.^7,14,26,27^ In pursuit of a unified model to estimate thermal tolerance limits for ectotherms across different experimental conditions, Jorgensen and colleagues presented a thermal injury model.^28^ Their model is based on a static knockdown time *t*_L_s and the temperature-related injury accumulation rate *R*. Given that the injury is the product of exposure duration and the injury accumulation rate (which increases exponentially with temperature), the model can be applied to static and dynamic temperature exposures. This approach is closely related to the ecotoxicological damage model in the GUTS framework. In the GUTS framework, damage is an abstract concept used to represent aspects of toxicodynamics. This approach relies on the existence of a threshold for effect, i.e., as long as the internal damage level is below a threshold, there are no effects of the chemical on mortality.^29^

In this study, we translate the damage model, as used for chemical effects in GUTS, to a damage model for the effects of temperature and, as such, create a TK–TD model for the interpretation of temperature effects. The parallels between the GUTS concept and the injury model by Jørgensen and colleagues^28^ and the mathematical derivation of the temperature damage model are presented in the Supporting Information (S01 and Table S1). After model calibration based on experimental data for the freshwater amphipod *Gammarus pulex*, model simulations were conducted for constant temperatures, daily temperature fluctuations, and heatwaves, deepening our understanding of the temperature as a stressor presenting itself in various environmental scenarios. With this, we lay the foundation to incorporate temperature as an additional stressor alongside chemicals in mechanistic effect models.

## 2 Methods

### 2.1 Temperature Damage Model Concept and Assumptions

**Figure 1:**
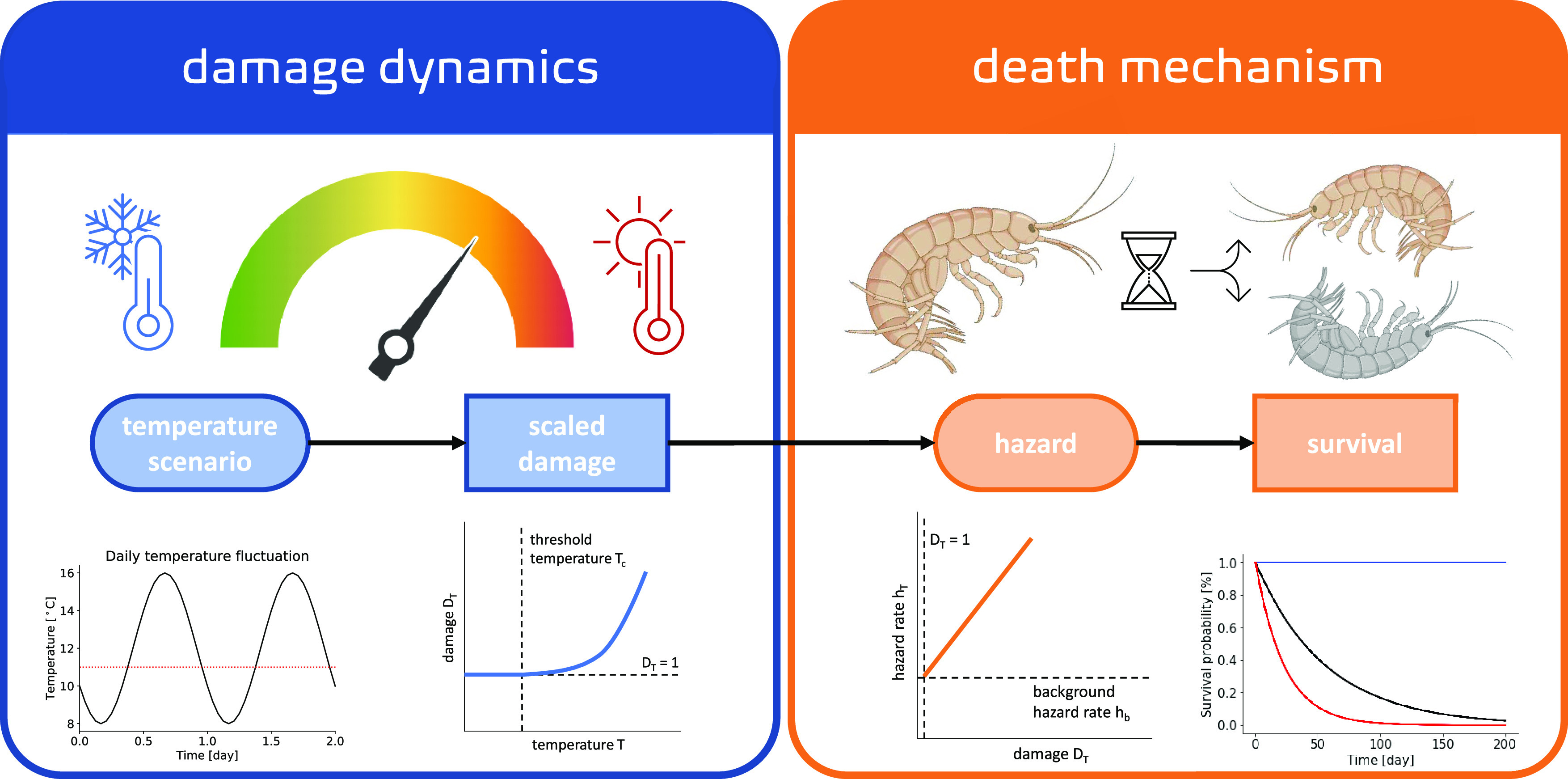
Conceptual representation of the temperature damage model. Model elements grouped in damage dynamics in blue (left box) and death mechanism in orange (right box) are accompanied by visual representations in the top panel and an example data representation in the lower panel. The model state variables (i.e., scaled damage and survival probability) are in squares, while the ellipses represent the temperature scenario (as a forcing variable) and the hazard rate (as integration from damage levels). Created with BioRender.com

The temperature damage model (Figure 1) comes with a set of assumptions, which for consistency, were phrased to relate closely to the assumptions made in the GUTS approach used to model the TK–TD of chemicals.^29^ The assumptions related to the damage dynamics comprise that the accrual flux of damage depends on the surrounding temperature, i.e., experimental water temperature when it exceeds a critical temperature, and that the repair flux is proportional to the damage level. Furthermore, the surrounding temperature is not influenced by damage accruing in the organism, and damage is treated as one homogeneous (well-mixed) compartment. It is important to note that the damage described here is not a measurable endpoint but rather a latent variable. Thus, we assume that the accrual or cumulation and the repair of such damage exist, but they cannot be measured directly. We diverge from the GUTS method where damage is scaled to the units of the exposure concentration by leaving damage unitless in our model (Table 1). Due to the assumption of exponential damage dynamic (eq 1), it is not possible to scale damage to the unit of temperature (see reasoning in Supporting Information, S01).

**Table 1:** Temperature Damage Model Variables, Parameter Symbols, and Explanations^a^

symbols	explanation	unit	value	95% CI
Variables				
*D*_T_	damage due to temperature	[–]	eq 1, with *D*_T_(0) = 1	
*h*_T_	hazard for individual in temperature damage model	day^–1^	eq 2	
*S*_T_	survival probability for individual in temperature damage model	[–]	eq 3, with *S*_T_(0) = 1	
*T*	absolute temperature (here: water temperature)	K	forcing variable (model input)	
Parameters (AIC = 282, *R*^2^ = 0.9521, NRMSE = 0.0287)				
*k*_T_	dominant rate for temperature-related damage accrual and/or temperature-related damage repair	day^–1^	5.76	0.322–100^b^
α	scaling parameter for the temperature effect	K^–1^	0.033	2.97^–05^–0.287
*T*_c_	critical temperature where damage accumulation starts	K	284.15	set value
*h*_b_	background hazard rate	day^–1^	0.004	2.88^–04^–0.018
*b*_T_	killing rate temperature	day^–1^	0.127	0.001–100^b^

a The respective model equation and starting value are provided for the model variables. Best-fit values and 95% confidence intervals are provided for each parameter, where appropriate.

b Boundary of the parameter space explorer.

Regarding the death mechanism, we assume that the damage level drives the temperature effect. Each individual organism has a damage level threshold. The value for this threshold is assumed to be the same for individuals of a species and can be chosen based on a priori knowledge and the optimal rearing temperature. When the damage level is below the threshold, there is no effect of the temperature on mortality. When damage exceeds the threshold value, the hazard rate due to the temperature stress becomes proportional to the value of the damage above the threshold.

Further, the background mortality is independent of the mortality caused by the temperature. To achieve this independence, the temperature conditions for the control group defining the background mortality must be set up at a temperature where the species is under optimal conditions. The background hazard rate can be constant for short temperature exposure tests. Finally, we assume that the organism does not change over time; in other words, the model parameters remain constant.

### 2.2 Mathematical treatment

A detailed description of the derivation of the new temperature damage model equations (derived from the injury model as presented in Jørgensen et al.,^28^ and the GUTS damage model shown in Jager and Ashauer^29^) is offered in the Supporting Information (S01). The temperature damage model assumes that the damage due to temperature (*D*_T_) increases when the temperature exceeds a critical temperature (*T*_c_). Above *T*_c_, the damage accumulates exponentially dependent on temperature, with a temperature coefficient of α. Below *T*_c_, any existing damage will be repaired. Note that we hereby assess only heating stress explicitly, not cold extremes. As we cannot measure the damage directly, we defined it as a dimensionless variable and defined arbitrarily that there is no damage if D_T_ equals one (eq 2). Damage accumulation and repair are modeled as a first-order process (eq 1) with a dominant rate of *k*_T_. The hazard rate (*h*_T_) is proportional to the dimensionless damage with a coefficient of *b*_T_ (eq 2). Finally, the survival function (*S*_T_(*t*)) defines the probability that the individuals survive until a certain time. This function is dependent on both the hazard rate due to temperature and other causes of death, such as background mortality (*h*_b_) (eq 3). All temperature model parameters, their units, and the calibrated values are listed in Table 1.
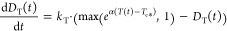
1
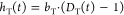
2
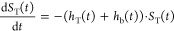
3

### 2.3 Model Calibration and Simulations

The model equations were implemented in the Bring Your Own Model (BYOM) modeling platform (www.debtox.info/byom.html, version 6.2) using MATLAB 2021b to perform all calculations. The model scripts are accessible at GitHub (https://github.com/NikaGoldring/Temperature-GUTS).

Experimental data of the control group from Henry and colleagues were used for model calibration.^12^ Briefly, they exposed 10 *G. pulex* individuals to each of the four different constant temperatures (10, 15, 20, 25 °C) over 8 days in three replicates, checking for mortality twice per day. They observed decreasing survival with an increasing temperature. Using these measured survival data over time, the model parameters and their confidence ranges were estimated using the parameter space explorer.^30^ Based on samples of the parameter space explorer, confidence ranges of model curves were created.

As discussed by Jørgensen et al., the real *T*_c_ of most species is unknown and will also likely depend on biological factors such as acclimation, age, sex, diet, etc.^28^ Thus, following the recommendation of Jørgensen et al. to choose a value by considering the rearing temperature (which was 15 °C in the experiment of Henry et al.^12^) and the evidence from their experiments where 10 °C did not show significant effects on survival, we chose a value of 11 °C as the set value for *T*_c_. Further, calibrating *T*_c_ based on the available data was not possible. Using a fixed threshold parameter contrasts with the GUTS model approach, where the threshold parameter is estimated from the available effect data. We also calibrated the model with a *T*_c_ set to 14 °C as a sensitivity assessment of this parameter, resulting in a similar model fit (Supporting Information, S02).

The model has the option to include background mortality h_b_ but in this study, we set this to zero due to data limitations. This implies that all hazard is caused by the effects of temperature. Model simulations investigated the predicted temperature effects under different temperature scenarios. Three categories were analyzed to cover scenario types applicable to the laboratory conditions: (1) constant temperatures, and more realistic environmental conditions; (2) daily temperature fluctuations; and (3) heatwave. Furthermore, a detailed analysis of different heatwave (HW) scenarios was conducted to investigate the relationship between the HW duration and intensity. For this, simulations with varying combinations of these factors were performed, and the survival probability was used as an evaluation variable for comparison between temperature scenarios. The ranges for tested HW duration (i.e., 7–100 days) and intensities (i.e., 3–6 °C) were chosen considering the assessment made by Woolway and colleagues predicting average lake HW intensities based on the RCP scenario 8.5 (representative concentration pathway, i.e., business as usual).^31^ For the heatwave scenario simulations, the daily temperature fluctuations of 4 °C around the average of 12 °C were increased by the intensity and each heatwave started at day 10.

## 3 Results

### 3.1 Calibration

**Figure 2:**
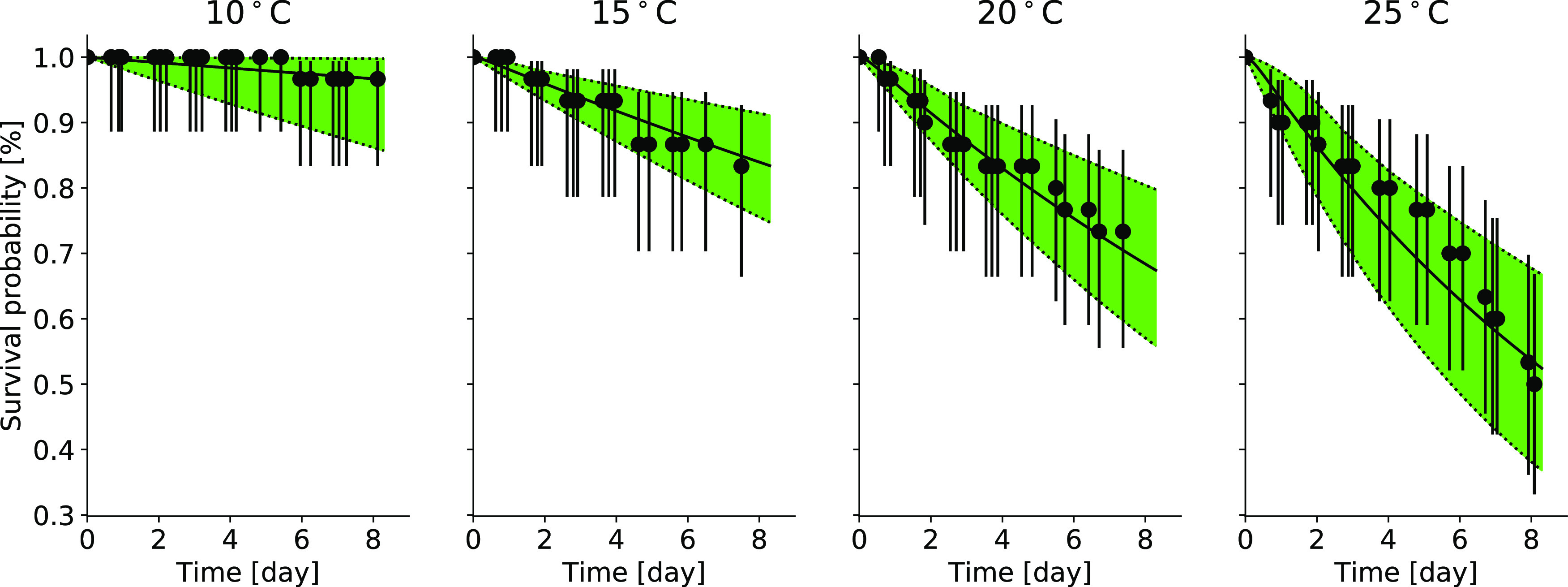
Model calibration of the survival probability for *G. pulex* over time. Solid lines show the model for the respective exposure scenarios (i.e.,10, 15, 20, 25 °C), and dotted lines represent their lower and upper confidence intervals. The experiments’ mean measured survival is plotted as dots with their Wilson score. For this calibration, *T*_c_ was set to 11 °C. Source of original experimental data: Henry et al.^12^

The experimental data of *G. pulex* exposed to different constant temperatures were fitted well by the temperature damage model during the model calibration. Across all temperature scenarios, the measured survival probabilities decreased with increasing temperature. The measured data matched well with the calibrated model (*R*^2^ = 0.9521, NRMSE = 0.0287) and lay within the 95% confidence intervals (Figure 2). However, the confidence limits of some parameters were very large (Table 1). The parameter space plot for the calibration shows well-defined boundaries for the background mortality (*h*_b_) and α but reveals possible identifiability problems for the dominant rate (*k*_T_) and the killing rate (*b*_T_), both missing a well-defined upper limit (Figure S1).

### 3.2 Model Simulations for Different Temperature Scenarios

**Figure 3:**
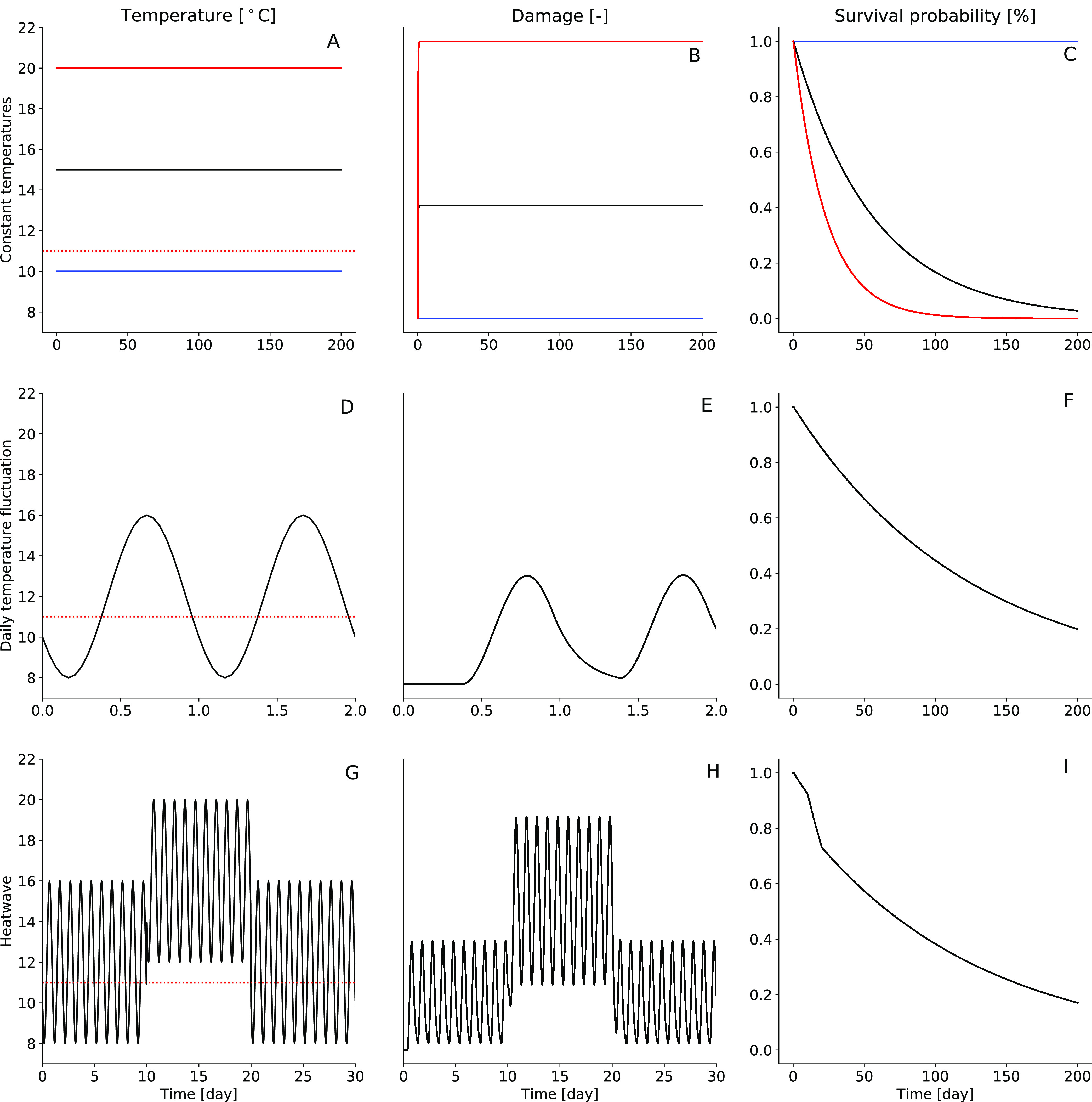
Model simulations of damage and survival probability for different scenario types. The different temperature scenario types are constant temperature scenarios (top row), daily temperature fluctuation (middle row), and daily temperature fluctuations with heatwaves (bottom row). *T*_c_ at 11 °C is marked with a dotted horizontal line. NOTE: While the survival probability is plotted for the whole simulation time (i.e., 200 days), the temperature and damage are plotted only for a representative period of the simulation (i.e., 2 and 30 days) for the daily temperature fluctuation and heatwave scenarios. Simulations were performed with *h*_b_ = 0.

Model simulations for the constant exposures to different temperatures (Figure 3A) showed a steep increase in the damage to its maximum (Figure 3B), where the highest temperature had the highest maximum damage. The damage and the survival probability (Figure 3C) for the 10 °C scenario did not change as the applied temperature was below *T*_c_. The survival probability for 15 and 20 °C decreased over time with a steeper decrease for the higher temperature. For the daily temperature fluctuation scenario (Figure 3D), the damage increased sharply during the periods where the temperature exceeded *T*_c_, reached its maximum, and decreased as quickly again when the temperature dropped below *T*_c_ (Figure 3E). The resulting survival probability decreased over time (Figure 3F). When applying the daily temperature fluctuations and adding a heatwave of a period of 10 days and a temperature increase of 4 °C at day 10 (Figure 3G), the damage showed the same dynamics as for the daily temperature fluctuation scenario and increased to a higher maximum during the heatwave period (Figure 3H). In the predicted survival probability, this is reflected in a sharp decrease during the heatwave period, after which the slope recovers to the same level as before (Figure 3I).

**Figure 4:**
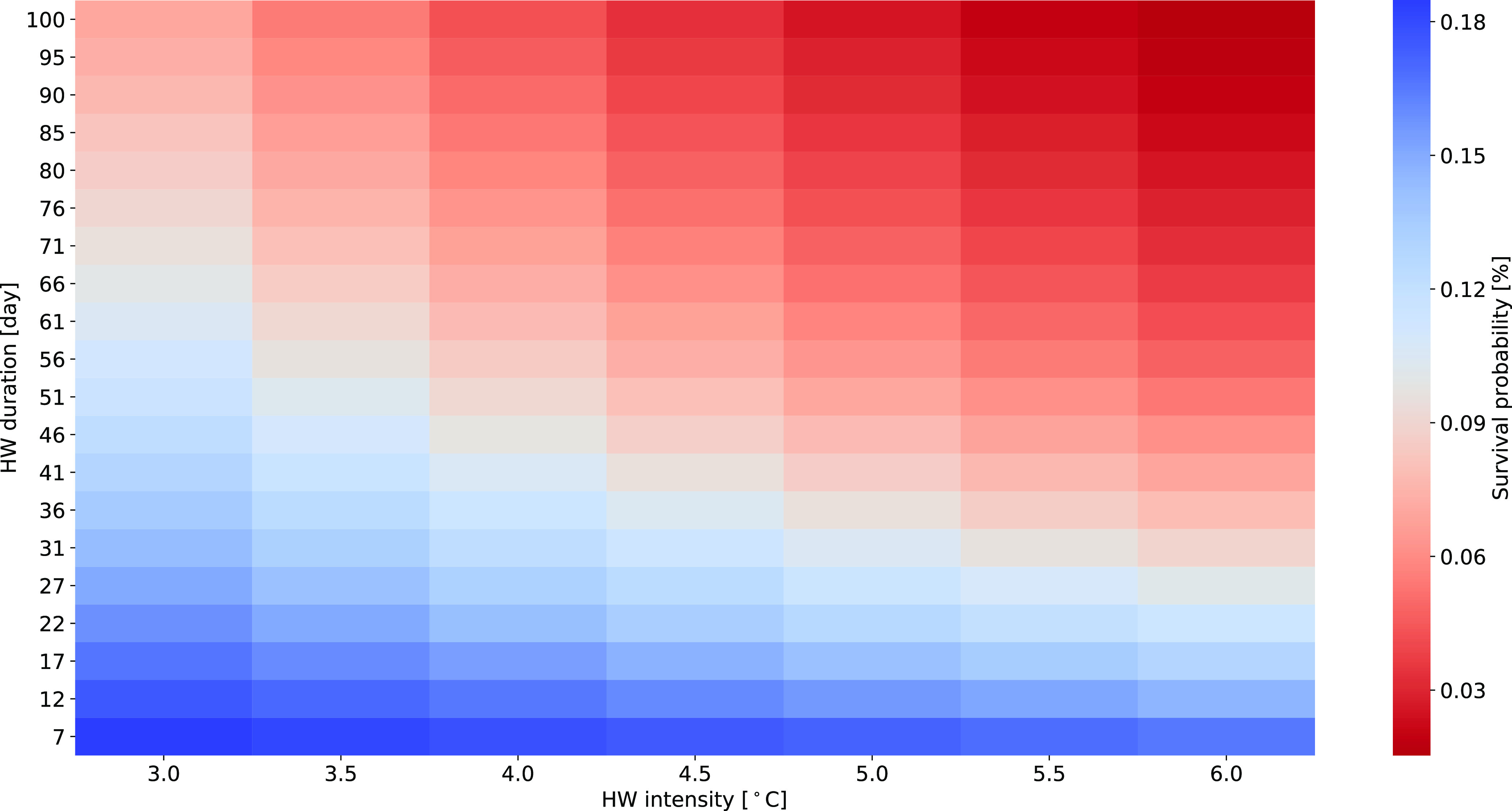
Heatmap for the survival probability at the end of the simulation period (*t* = 200 days) depending on different heatwave intensities and durations. During the heatwave, the base water temperature (daily temperature fluctuations of 4 °C around the average of 12 °C) was increased by the intensity. For each scenario, the heatwave starts on day 10. Simulations were done with *h*_b_ = 0.

When testing different combinations of heatwave intensity (i.e., from 3 to 6 °C) and durations (i.e., from 7 to 100 days), the respective survival probability at the end of the simulation period was plotted in a heatmap (Figure 4). The figure shows the nonlinear effect of temperature, where a higher intensity has more effect than a longer duration. Clearly, high intensities combined with long durations result in the lowest survival probability.

## 4 Discussion

The temperature damage model presented in this study combines two models: the relatively new thermal injury model, as explained by Jørgensen et al.,^28^ and the well-established GUTS.^29^ The most important features we added to the concept of the thermal injury model are to allow for injury or damage recovery, which was not represented before, and to relate the equations to the GUTS framework by including the hazard function, which allows fitting the model on survival data. Both features will be essential for future use in environmental risk assessment. To allow for damage recovery, it is necessary to simulate realistic temperature scenarios, where temperatures vary from harmful to harmless conditions. Further, implementing the hazard function will facilitate coupling the temperature damage model in a multiple-stress scenario with a chemical stressor modeled in GUTS.

Based on our visual judgment and the quantitative model efficiency measures (*R*^2^ = 0.9521, NRMSE = 0.0287), the effect of temperature on *G. pulex* survival, as measured by Henry et al.^12^ was captured accurately in the model calibration of our new temperature damage model (Figure 2). Based on the goodness of fit parameter values, the calibration result also complies with the criteria laid down by the EFSA scientific opinion on TK–TD modeling.^32^

As frequently observed in model calibrations of the GUTS-RED parameters,^33^ we found a high value for the dominant rate *k*_T_, suggesting fast kinetics (Table 1). However, due to data limitations, the confidence limits were very large, so based on our data we cannot be sure this damage accrual is really a fast process. It seems however not unreasonable to expect relatively fast dynamics, as ectotherms will quickly take their environmental temperature, and the most dominant effect of high temperatures is a decrease of protein stability.^34^ Enzymes unfold above a certain temperature (the melting temperature), which leads to declines in enzymatic rates. If the kinetics are indeed fast, this might also explain why other parameters (here: *b*_T_) also run into the boundaries of the parameter space explorer (Figure S1). This is also frequently found in the calibration of the related GUTS-RED model (discussed in Jager 2020,^35^ chapter 4). This implies that the effect of the temperature on mortality is fast. The model cannot distinguish between “fast” and “very fast”. Therefore, a wide range of *b*_T_ values gives an equally good fit to the data, where eventually, *b*_T_ can converge against the set boundary value (or infinity). As argued for GUTS-RED models elsewhere,^36^ one or two additional observations on mortality early in the experiment would have probably made a difference in pinpointing the value of *b*_T_.

Looking at the model simulations for different scenarios (Figure 3), we observe fast damage dynamics, as it is driven by a high value for *k*_T_ (5.76 day^–1^). This implies that when the temperature threshold is exceeded, effects on mortality can be seen within 1 day. On the other hand, when the temperature falls below the threshold, effects on mortality also disappear within 1 day (Figure 3I). In the context of heatwave simulations, it appears that the intensity of the heatwave has a stronger effect on the survival probability than its duration (Figure 4). However, as expected, the combination of high intensities and long durations resulted in the highest effect on survival. Overall, the model can be used to predict the effect of complex temperature scenarios, including the nonlinear effect of heatwaves and daily temperature fluctuation.

### 4.1 Model Assumptions and Limitations

Like every model, the temperature damage model is based on assumptions, simplifying the complex reality that it represents. For this model, the most crucial assumption centers around the threshold for the temperature effect (*T*_c_). In the chemical GUTS-RED-SD approach, the threshold is determined as the value of the external concentration that does not affect the survival probability. In the temperature damage model, however, we currently do not calibrate *T*_c_, but we fix *T*_c_ based on biological knowledge (see arguments in Section 2). Furthermore, in the chemical model, the background mortality is based on the control treatment where no chemical stressor is assumed to be present. The difficulty with the temperature effect modeling is that it is unclear under which temperature conditions no effects on survival will occur since there is nothing like a nonexposed experiment. For effect modeling, observations in control experiments are of crucial importance for parameter estimates. Choosing the optimal temperature condition for the control is hence crucial for estimating the effect of temperature on survival. If the control treatment already impacts survival, then the overall effect of temperature will be underestimated.

Another central assumption of the presented model is the assumption of temperature damage as a latent state variable for the actual processes affected by the temperature, causing damage in the organism. As potentially every physiological process is governed by temperature, it seems unlikely to quantify the actual damage done by a certain temperature for each process (i.e., damage to macromolecules, impact on functioning, or denaturation of enzymes). Consequently, we choose the approximation of an overall temperature damage as presented in this study, similar to the approach used in the chemical GUTS approach.^29^ In contrast to chemical GUTS, the damage calculated here is not scaled to a concentration but remains dimensionless (Table 1). This makes it even more difficult to interpret the damage state in a meaningful way. At the same time, it still enables an insight into the damage dynamics in relation to the applied temperature scenario. In our simulations, we observed that damage accumulation is driven by the external temperature dynamics above *T*_c_ and that it recovers in the periods where the temperature is below *T*_c_ (Figure 3E).

Currently, no critical temperature is defined at the lower side of the temperature preferendum, while in reality, those conditions will also be stressful.^14,37^ However, most species are adapted to low temperatures by overwintering strategies,^38^ and some species can even resist freezing.^39^ At low temperatures, all metabolic rates slow down according to the Arrhenius equation, making the effect of low temperature more gradual.^40^ Determining these effects would require experimental data that include the full range of temperature scenarios to capture both ends of the temperature performance curve.

Another obstacle with defining species’ thermal tolerances on both sides (i.e., lower and upper temperature limits) is that they are different between populations or even with the organism life-stage due to phenotypic plasticity.^7,26,41,42^ The acclimatization of organisms has been shown to alter their thermal performance curves^6,43^ and their sensitivity to chemical stressors.^44^ The data set used here was based on *G. pulex*, originating from a relatively cold stream (9 °C), hence validating the current calibrated model with survival data of *G. pulex* from different locations will likely fail. When validating the temperature damage model, adjustments to the threshold parameter (*T*_c_) are likely to be needed to account for differences in life stages and their life histories regarding acclimatization temperatures. However, looking at this from another perspective, using data from different populations could facilitate investigating intraspecies sensitivity differences. In any case, while these circumstances should be considered for model calibration, validation, and application, they do not undermine the potential of the temperature damage model to be a useful tool to study the effect of temperature in separation from chemical stressors.

### 4.2 Relevance for Environmental Risk Assessment of Chemicals

As previously shown in empirical studies, temperature itself can influence organisms’ survival, depending on the actual experienced temperature and the duration of exposure.^45^ In this case, we considered temperature as an additional stressor for the individual rather than a modulating factor of chemical stressors. Although studies have investigated the combined effects of temperature and chemicals,^10,13,22,46^ currently, temperature is not implemented as an additional stressor in chemical risk assessments. With extreme events projected to increase in their frequency and magnitude,^31,47,48^ we saw the need to develop a novel temperature damage model with the goal of including temperature as an additional and modulating stressor into TK–TD models for chemicals. To achieve this multiple-stressor assessment through mechanistic modeling, the presented temperature damage model should be combined with the chemical GUTS-RED following the approach previously used for mixture toxicity.^49,50^ The first and simplest option is to assume independent action of temperature and chemical effects, hence adding hazards due to the temperature and chemicals. However, beyond this most obvious option, identifying the interaction type (i.e., synergistic or antagonistic), testing which stressor is driving the interaction, and finally simulating and predicting the combined effect has already been successfully demonstrated in the GUTS mixture model.^49^

Furthermore, a combination of the new temperature damage model and the GUTS-T approach, where temperature modulates the chemical parameters, should be explored.^17^ Considering the spectrum of temperature performance curves, it seems likely that temperature acts as both a modulating factor and a stressor across the whole temperature spectrum. Thus, combining the newly developed temperature damage model with GUTS mixture and GUTS-T seems a promising approach to gain insights into the mechanistic interactions of temperature and chemical stress. This ultimately enables us to investigate the effects of chemicals in current and future climate scenarios and thus supports a realistic and protective risk assessment for chemicals in the environment.

## Supporting Information Available

Derivation of the model equations. Model calibrations (PDF)
