# Supplementary material for: Toxicokinetic–Toxicodynamic Model to Assess Thermal Stress
Source: Environ Sci Technol. 2023 Dec 8;57(50):21029–37. doi: 10.1021/acs.est.3c05079 (PMC10734255; doi:10.1021/acs.est.3c05079)
Supplement: Supplementary file 1 — es3c05079_si_001.pdf [file es3c05079_si_001.pdf]

Supporting Information for the article:

A toxicokinetic-toxicodynamic model to assess thermal stress

Authors:

Annika Mangold-Döring[1,2]*, Jan Baas[2], Paul J. van den Brink[1,2], Andreas Focks[3], Egbert H. van Nes[1]

*annika.mangold-doering@wur.nl

Affiliations:

[1]Department of Aquatic Ecology and Water Quality Management, Wageningen University and Research, P.O. box 47, 6700 AA Wageningen, the Netherlands

[2]Wageningen Environmental Research, P.O. Box 47, 6700 AA Wageningen, the Netherlands

[3]System Science Group /Institute of Mathematics, Osnabrück University, Barbarastr. 12, D-49076 Osnabrück, Germany

Tables: 1

Figures: 14

S01 Derivation of the model equations

Here we describe the development of the temperature damage module presented in this study, starting from the state-of-the-art damage module for chemicals as part of the widely used General Unified Threshold model of Survival (GUTS). The previously described GUTS approach (Ashauer et al. 2011; Jager and Ashauer 2018) simulates the probability of death of individuals over time-based on measured survival data. As proposed by Jager and Ashauer, the mechanistic approach of GUTS, though developed and increasingly used for chemical risk assessment, can be applied to other stressors that cause effects on survival (Jager and Ashauer 2018).

We perform a systematic analysis of the dimensions of the damage equation of the GUTS model (equation S1) and damage in our temperature approach (equation S7). The purpose of such analysis is to eliminate parameters that are not identifiable because they have the same effect on the model as another parameter. For instance if we want to do a regression of the following equation we cannot identify both the parameters a and b using data of the variables Y and X, because the model is overparameterized:

Y=a*b*X + c

Similarly, if variable Z is a latent variable that cannot be measured, we cannot determine parameter b in the following model because this parameter is dependent on the scale of Z:

Y=a*X+b*Z+c

In our approach, we cannot measure damage, so our purpose is to eliminate parameters that can only be determined if we would be able to quantify damage.

Derivation example based on the damage equation of the GUTS model

For the damage module in GUTS, a one-compartment model is assumed with first-order kinetics (eq. S1). Here, the damage accrual is proportional to the external water concentration of the chemical (C_w), and the damage repair is proportional to the amount of damage (D). With those state variables and the damage accrual rate (k_a) and the damage repair rate (k_r), we can describe the damage dynamics for chemicals over time (t) with eq. S1.

$$\frac{dD\ (t)}{dt} = k_a \cdot C_w(t) - k_r \cdot D\ (t)$$ eq. S1

By applying the method of Edelstein-Keshet (1988, page 126-127) that aims to make all state variables non-dimensional, we replace *D(t)* in eq. S1 with $D^*\widehat{D}$, where D^* is the new non-dimensional state variable and $\widehat{D}$ is a quantity that represents the units of measurement of *D(t)* (Edelstein Keshet, 1988). Similarly we also replace the chemical substance $C_w(t) = C_w{}^*\widehat{C_w}$ and time $t = t^*\,\tau$ $(where\ C_w{}^*$ and t^* are dimensionless variable and $\widehat{C_w}$ and τ are parameters that carry the dimensional scale):

$$\frac{d\ D^*\widehat{D}}{d\tau\ t^*} = k_a\ C^*\hat{C} - k_r\ D^*\widehat{D}$$ eq. S2

Dividing both sides of the equation by $\widehat{D}$ and multiplying with τ:

$$\frac{d\ D^*\widehat{D}\ \tau}{d\tau t^*\ \widehat{D}} = \frac{k_a}{\widehat{D}}\ \tau\ C_w{}^*\widehat{C_w} - k_r\ \tau\ D^*\frac{\widehat{D}}{\widehat{D}}$$ eq. S3

Simplifying and factoring $k_r\,\tau$ out:

$$\frac{dD^*}{dt^*} = k_r\,\tau\left(\frac{k_a}{k_r\,\widehat{D}} C_w{}^* \widehat{C_w} - D^*\right) \qquad \text{eq. S4}$$

Jager and Ashauer (2018) made at this point the following choices: they did not change the dimensions of time t and C_w, i.e., τ=1 and $\widehat{C_w} = 1$, but removed k_a from the equation by choosing:

$$\widehat{D} = \frac{k_a}{k_r} \qquad \text{eq. S5}$$

We then arrive at the following model (in which we dropped the *).

$$\frac{dD(t)}{dt} = k_r\,(C_w(t) - D(t)) \qquad \text{eq. S6}$$

Because we chose $\widehat{C_w} = 1$, this means that $D(t)$ should have the same dimensions as $C_w(t)$. Therefore, the damage as presented in the GUTs model is scaled to the chemical concentration and consequently, the unit of $\widehat{C_w}$.

Derivation of the damage equation of the temperature damage model

The temperature damage model presented in this study was inspired the temperature injury model as presented by Jørgensen et al. (2021). With the aim to develop "a unifying model to estimate thermal tolerance limits in ectotherms across static, dynamic, and fluctuating exposures to thermal stress", their model enables the cross-study comparison of thermal tolerance measures. Their model is constructed of similar parts to the GUTS model (Table S1). However, it comes with its own limitations. The model of Jørgensen et al. (2021) only considers fixed temperatures or a linearly increasing temperature, and ignores damage repair. This considerably limits the use of this model to be applied on realistic temperature scenarios, varying from temperatures in- and outside of the temperature niche of the organism. We addressed these limitations in the temperature damage model by using a structure closely related to the damage equation of GUTS. In the temperature damage model, the damage accrual depends on the external temperature condition over time, expressed as some function g(T(t)). Thus our starting point is, to replace the chemical concentration as the stressor in eq. S1 with this temperature stress function (g(T(t)).

$$\frac{d\mathrm{D}_{\mathrm{T}(t)}}{dt} = k_a \cdot \mathrm{g}(\mathrm{T(t)}) - k_r \cdot \mathrm{D}_{\mathrm{T}(t)} \qquad \text{eq. S7}$$

Jørgensen et al. (2021) argue that temperatures' effect on survival is best described by an exponential relationship. There is namely much evidence that time to death (or knock-down time) increases exponentially with increasing temperature. This is first shown by (Bigelow 1921) for bacteria. This observation is frequently confirmed for other species in the scientific literature literature (Schulte, Healy, and Fangue 2011; Sutcliffe, Carrick, and Willoughby 1981; Mundim et al. 2020; Jørgensen, Malte, and Overgaard 2019; Aquilanti et al. 2010). Moreover, we know that within the temperature niche of the organism, there is no damage caused by temperature to be expected. Thus, we should define a threshold temperature T_c above which temperature causes damage. Below this threshold, the temperature probably still modulates, as discussed elsewhere (Huang et al. 2023; Mangold-Döring et al. 2022; Schulte, Healy, and Fangue 2011).

Jørgensen et al. (2021) show that their approach indeed predicts an exponential thermal death-time curve. Thus, we decided to use the exponential expression of Jørgensen et al. as the function to express the influence of temperature-related damage accrual:

$$g(T(t)) = e^{\alpha(T(t)-T_c)} \qquad \text{eq. S8}$$

With this, equation S7 becomes

$$\frac{dD_T(t)}{dt} = k_a \cdot e^{\alpha(T(t)-T_c)} - k_r D_T(t) \qquad \text{eq. S9}$$

Again we substitute some new variables $D_T(t) = D_T{}^*\widehat{D_T}$, $T(t) = T^*\hat{T}$ and, $t = t^*\,\tau$

$$\frac{d\, D_T{}^*\widehat{D_T}}{d\,\tau\, t^*} = k_a \cdot e^{\alpha(T^*\hat{T}-T_{C*})} - k_r D_T \widehat{D_T} \qquad \text{eq. S10}$$

Dividing both sides of the equation by $\widehat{D_T}$ and multiplying with τ:

$$\frac{d\, D_T{}^*\widehat{D_T}\tau}{d\,\tau\, t^*\widehat{D_T}} = \frac{\tau\, k_a}{\widehat{D_T}} e^{\alpha(T^*\hat{T}-T_{C*})} - k_r \frac{\tau\, \widehat{D_T}}{\widehat{D_T}} D_T{}^* \qquad \text{eq. S11}$$

Which simplifies to:

$$\frac{d\, D_T{}^*}{d\, t^*} = \frac{\tau\, k_a}{\widehat{D_T}} e^{\alpha(T^*\hat{T}-T_{C*})} - k_r \tau D^*{}_T \qquad \text{eq. S12}$$

Factoring $k_r\tau$ out:

$$\frac{d\, D_T{}^*}{d\, t^*} = k_r\tau\,(\frac{k_a}{k_r\widehat{D_T}} e^{\alpha(T^*\hat{T}-T_{C*})} - D_T) \qquad \text{eq. S13}$$

Similar as the GUTS approach (Jager and Ashauer, 2018), we wish to keep normal units for time ($\tau = 1$) and temperature ($\hat{T} = 1$). With the following choice for $\widehat{D_T}$, we remove one parameter (k_a):

$$\widehat{D_T} = \frac{k_a}{k_r} \qquad \text{eq. S14}$$

Note that this is completely analogous to the GUTS approach, but in this case it implies that D_T is dimensionless, because as an e-power is dimensionless. Which gives the used equation (in which we dropped the * and renamed k_r to k_T):

$$\frac{dD_T(t)}{dt} = k_T\left(e^{\alpha(T(t)-T_c)} - D_T(t)\right) \qquad \text{eq. S15}$$

This new temperature-related damage model describes the effect on survival induced by stressful (in contrast to modulating) temperature conditions. To estimate the model parameters based on the measured survival, the rest of the model is defined as in the main script (eq. 1-3).

Table S1: Table to outline parallels in concepts (Jørgensen vs. GUTS). GUTS-RED parameters are used.

**Symbol pairs** (GUTS ~ Jørgensen)	**Function**	**GUTS explanation and unit**	**Jorgensen explanation and unit**
D_w ~ d	Damage/injury state variable	Damage level in an organism, scaled to external concentration	Accumulated injury/damage
C_w ~ T	Stressor state variables	Chemical exposure concentration, i.e., mol · L^{-1}	Temperature, i.e., K
m_w ~ R_0	Threshold for damage/injury accumulation	Median of the distribution of thresholds, concentration of chemical in the environment, i.e., mol · L^{-1}	Temperature-related injury accumulation rate that exactly matches the injury repair rate at T_c, unit of injury per time, i.e., t^{-1}
k_d ~ R	Damage/injury accumulation rate	k_d = dominant rate constant, per time (i.e., d^{-1})	Temperature-related injury accumulation rate, per time (i.e., h^{-1})

S02 Model calibration

Model calibration with T_c =11 °C

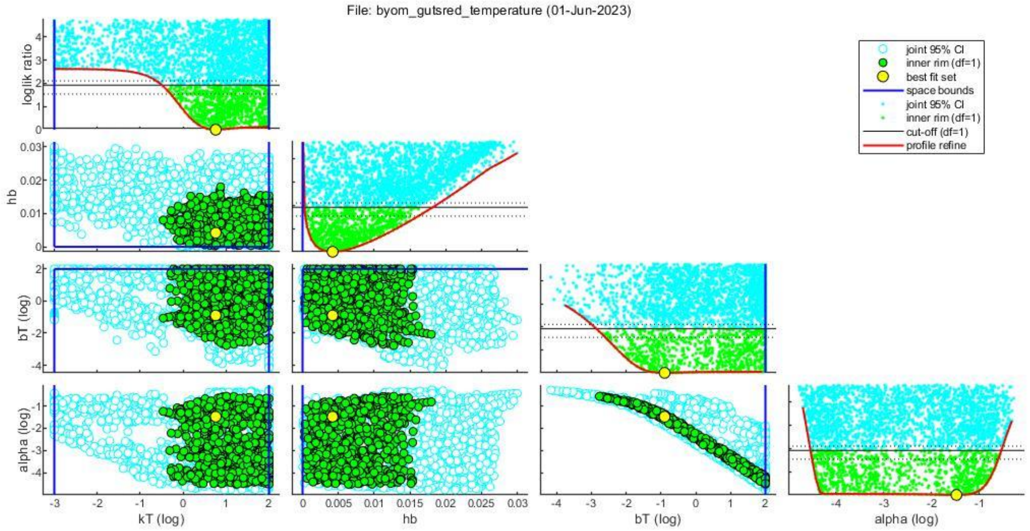

Figure S1: Parameter space plot, calibration (for T_c = 11 °C). The plots on the diagonal show the profile likelihoods for the individual parameters. The scatter plots underneath are the 95% joint confidence regions. The yellow dots mark the best-fit values for each parameter and green dots show the parameter sets within the critical value (i.e., under the horizontal black line). The parameter symbols and units can be obtained from Table 1 in the main script.

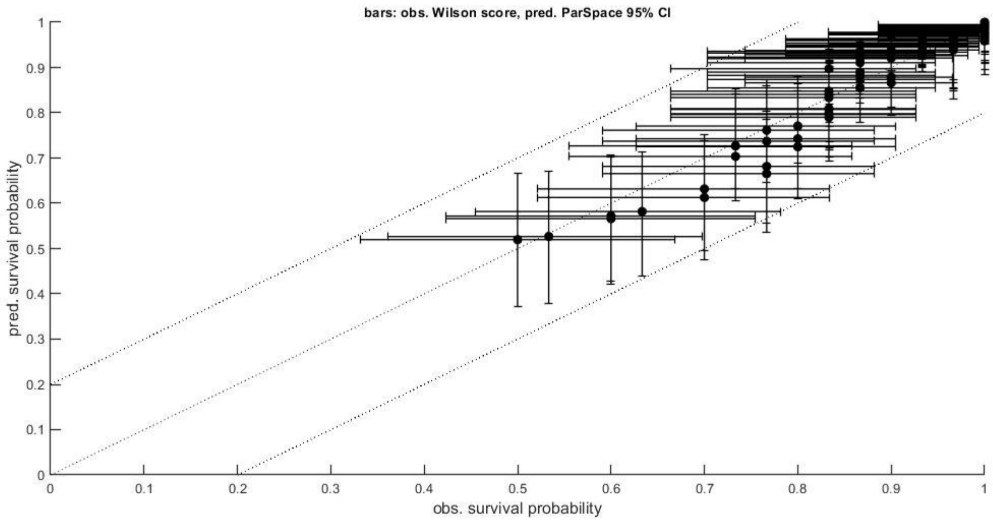

Figure S2: Predicted and observed survival probability for model calibration (for T_c = 11 °C). Vertical error bars represent the uncertainty of the model prediction (their 95% confidence intervals). Horizontal error bars are the Wilson scores intervals of the observed survival data.

Model calibration with T_c =14 °C

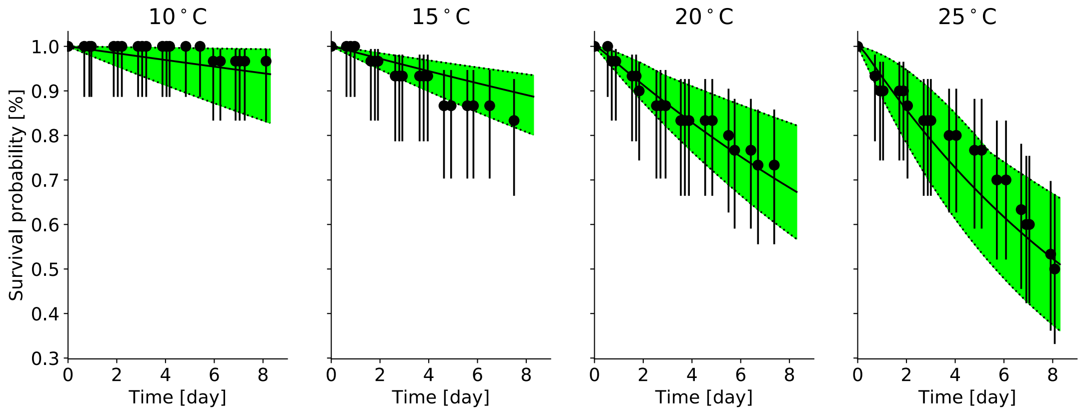

Figure S3: Model calibration of the survival probability for Gammarus pulex is plotted over time. Solid lines show the model for the respective exposure scenarios (i.e.,10, 15, 20, 25 °C), and dotted lines represent their lower and upper confidence intervals. The experiments' mean measured survival is plotted as dots with their Wilson score. For this calibration, T_c was set to 14 °C. Source for original experimental data: Henry et al., 2017

Table S2: Temperature damage model variable and parameter symbols and explanations. For model variables the respective model equation is provided, and for the parameters their best fit value along with their 95% confidence interval is provided. For this calibration, T_c was set to 14 °C.

Symbols	Explanation	Unit	Value	95 % CI
Variables				
D_T	Damage due to temperature	[-]	Eq. 1	
h_T	hazard for individual in temperature damage model	d^{-1}	Eq. 2	
S_T	Survival probability for individual in temperature damage model	[-]	Eq. 3	
T	Absolute temperature (here: water temperature)	K	Forcing variable (model input)	
Parameters (AIC = 284, R^2 = 0.9148 and NRMSE = 0.0383)				
k_T	dominant rate for temperature related damage accrual and/or temperature related damage repair	d^{-1}	6.56	0.009-100*
α	Scaling parameter for the temperature effect	K^{-1}	6.78^{-05}	3.46^{-05}-0.372
T_c	Critical temperature where damage accumulation starts	K	287.15	set value
h_b	background hazard rate	d^{-1}	0.008	0.001-0.022
b_T	killing rate temperature	d^{-1}	100*	0.001-100*

*Boundary of the parameter space explorer

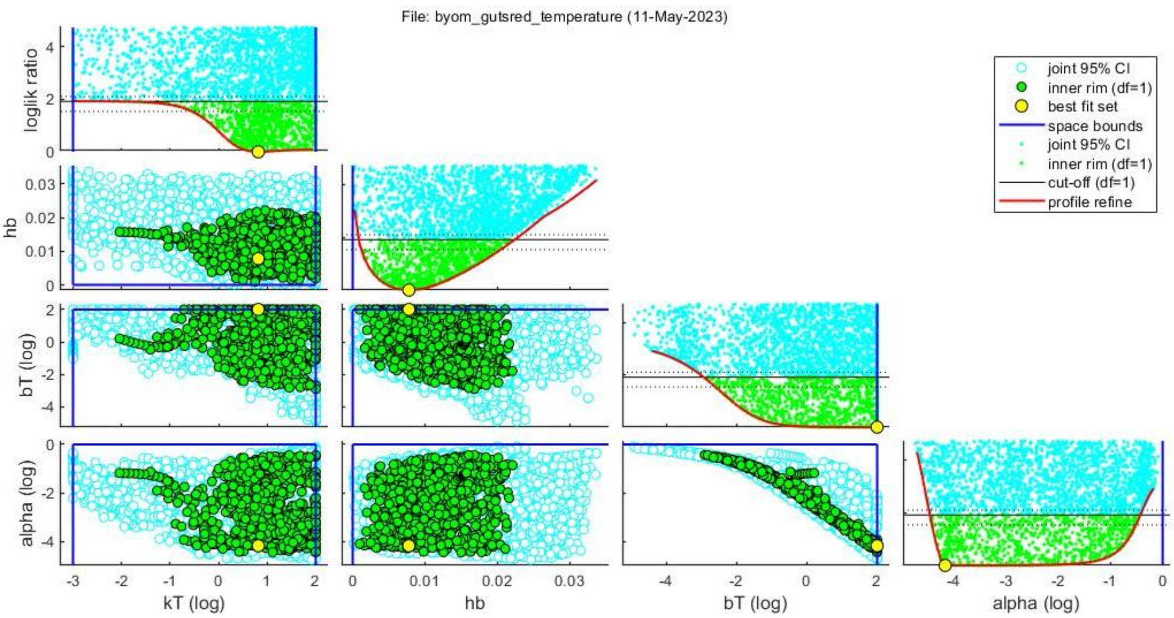

Figure S4: Parameter space plot, calibration (for T_c = 14 °C). The plots on the diagonal show the profile likelihoods for the individual parameters. The scatter plots underneath are the 95% joint confidence regions. The yellow dots mark the best-fit values for each parameter and green dots show the parameter sets within the critical value (i.e., under the horizontal black line). The parameter symbols and units can be obtained from Table 1 in the main script.

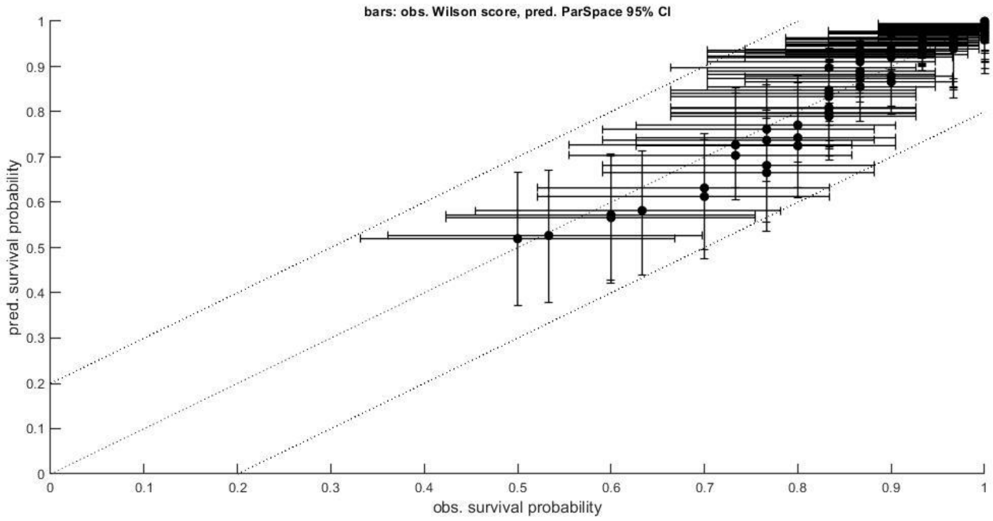

Figure S5: Predicted and observed survival probability for model calibration (for T_c = 14 °C). Vertical error bars represent the uncertainty of the model prediction (their 95% confidence intervals). Horizontal error bars are the Wilson scores intervals of the observed survival data.

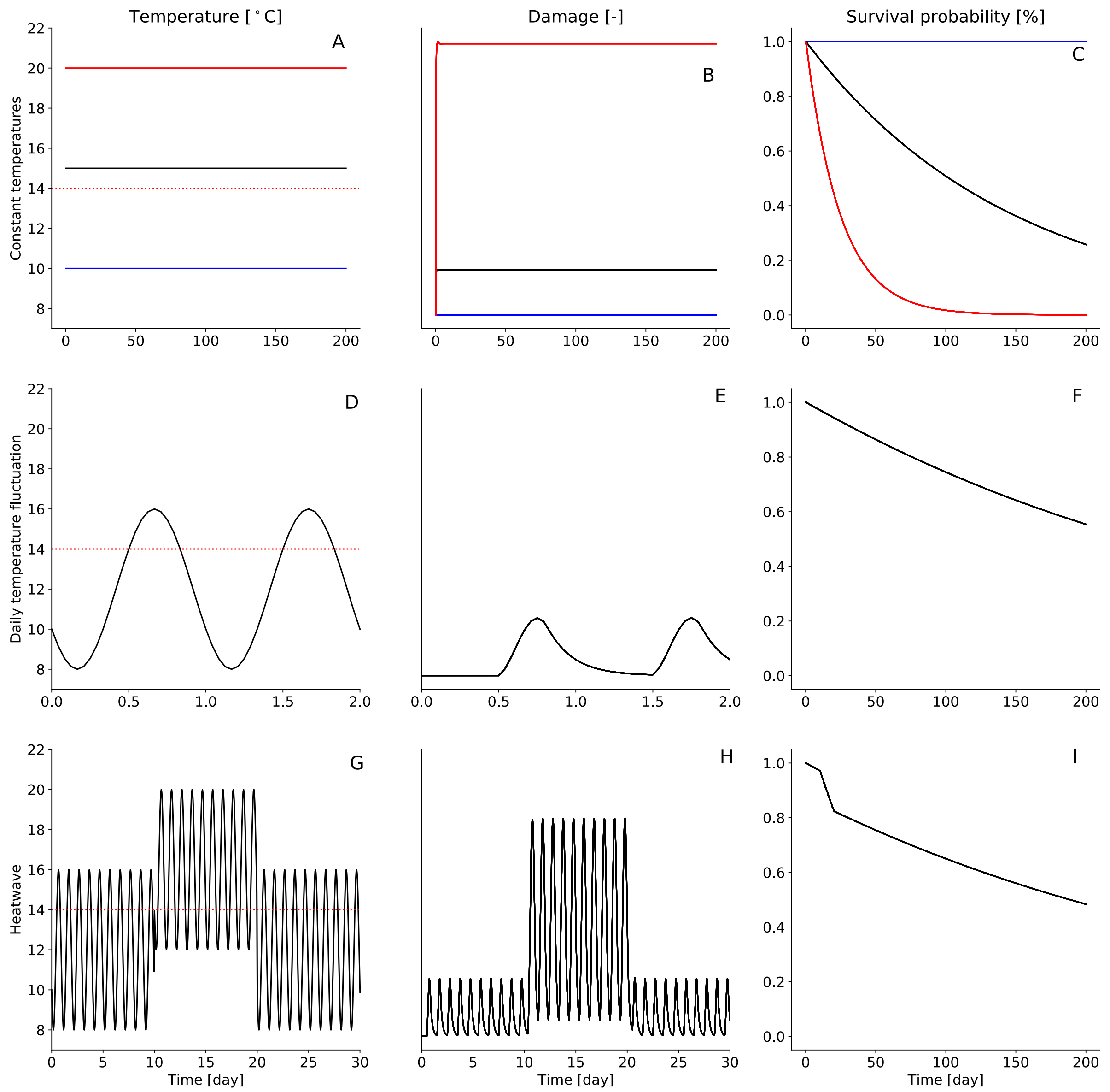

Figure S6: Model simulations of damage and survival probability for different temperature scenario types. The different temperature scenario types are constant temperature scenarios (top row), daily temperature fluctuation (middle row), and daily temperature fluctuations with heatwaves (bottom row). T_c with 14 °C is marked with a dotted horizontal line. NOTE: While the survival probability is plotted for the whole simulation time (i.e., 200 days), the temperature and damage are plotted only for a representative period of the simulation (i.e., 2 and 30 days) for the daily temperature fluctuation and heatwave scenarios. Simulations were done with h_b = 0.

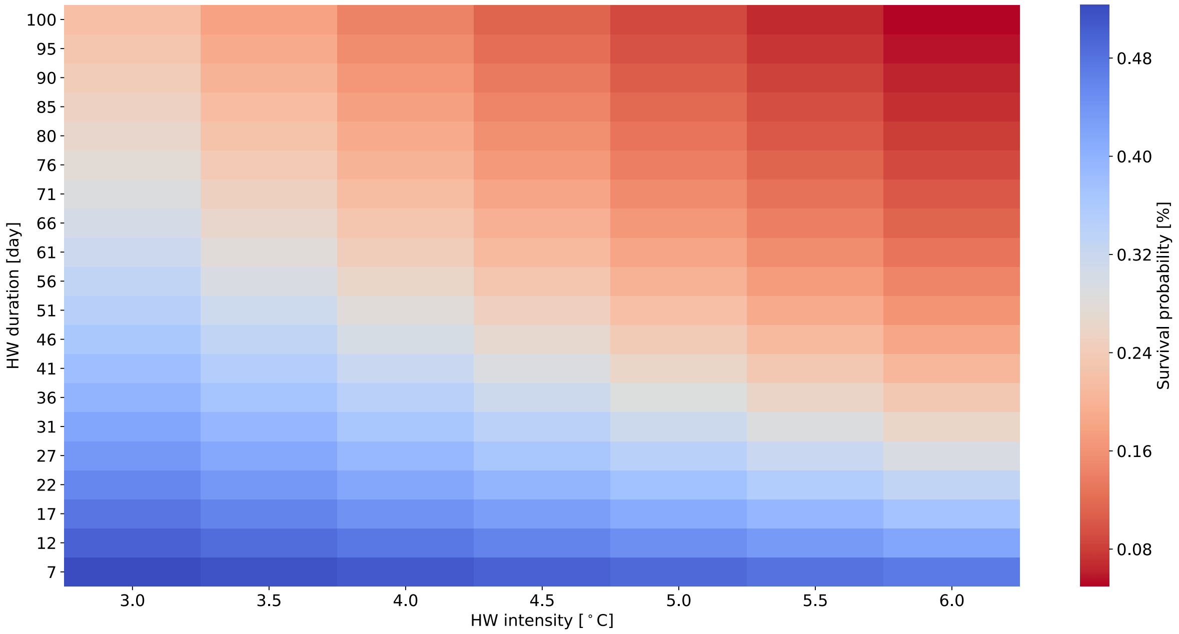

Figure S7: Heatmap for the survival probability at the end of the simulation period (t=200 days) depending on different heatwave intensities and durations. During the heatwave the base water temperature (daily temperature fluctuations of 4 °C around the average of 12 °C) was increased by the intensity. For each scenario, the heatwave start was at day 10. Simulations were done with h_b = 0 and T_c = 14°C.

References

Ashauer, Roman, Annika Agatz, Carlo Albert, Virginie Ducrot, Nika Galic, Jan Hendriks, Tjalling Jager, et al. 2011. "Toxicokinetic-Toxicodynamic Modeling of Quantal and Graded Sublethal Endpoints: A Brief Discussion of Concepts." *Environmental Toxicology and Chemistry* 30 (11): 2519–24. https://doi.org/10.1002/etc.639.

Bigelow, W. D. 1921. "The Logarithmic Nature of Thermal Death Time Curves." *The Journal of Infectious Diseases* 29 (5): 528–36. https://doi.org/10.1093/infdis/29.5.528.

Edelstein-Keshet, Leah. 1988. *Mathematical Models in Biology*. 1 edition. New York, NY: McGraw-Hill, Inc.

Henry, Y., C. Piscart, S. Charles, and H. Colinet. 2017. "Combined Effect of Temperature and Ammonia on Molecular Response and Survival of the Freshwater Crustacean Gammarus Pulex." *Ecotoxicology and Environmental Safety* 137 (March): 42–48. https://doi.org/10.1016/j.ecoenv.2016.11.011.

Huang, Anna, Annika Mangold-Döring, Huitong Guan, Marie-Claire Boerwinkel, Dick Belgers, Andreas Focks, and Paul J. Van den Brink. 2023. "The Effect of Temperature on Toxicokinetics and the Chronic Toxicity of Insecticides towards Gammarus Pulex." *Science of The Total Environment* 856 (January): 158886. https://doi.org/10.1016/j.scitotenv.2022.158886.

Jager, Tjalling, and Roman Ashauer. 2018. *Modelling Survival under Chemical Stress A Comprehensive Guide to the GUTS Framework*.

Jørgensen, Lisa Bjerregaard, Hans Malte, Michael Ørsted, Nikolaj Andreasen Klahn, and Johannes Overgaard. 2021. "A Unifying Model to Estimate Thermal Tolerance Limits in Ectotherms across Static, Dynamic and Fluctuating Exposures to Thermal Stress." *Scientific Reports* 11 (1): 1–14. https://doi.org/10.1038/s41598-021-92004-6.

Mangold-Döring, Annika, Anna Huang, Egbert H. van Nes, Andreas Focks, and Paul J. van den Brink. 2022. "Explicit Consideration of Temperature Improves Predictions of Toxicokinetic–Toxicodynamic Models for Flupyradifurone and Imidacloprid in Gammarus Pulex." *Environmental Science & Technology*, October. https://doi.org/10.1021/acs.est.2c04085.

Schulte, Patricia M., Timothy M. Healy, and Nann A. Fangue. 2011. "Thermal Performance Curves, Phenotypic Plasticity, and the Time Scales of Temperature Exposure." *Integrative and Comparative Biology* 51 (5): 691–702. https://doi.org/10.1093/icb/icr097.
